# Localization Based on Magnetic Markers for an All-Wheel Steering Vehicle

**DOI:** 10.3390/s16122015

**Published:** 2016-11-29

**Authors:** Yeun Sub Byun, Young Chol Kim

**Affiliations:** 1 Korea Railroad Research Institute, 176 Cheoldo Bangmulgwan-ro, Uiwang, Gyeonggi-do 16105, Korea; 2 Department of Electronics Engineering, Chungbuk National University, 1 Chungdae-ro, Seowon-gu, Cheongju, Chungbuk 28644, Korea; yckim@cbu.ac.kr

**Keywords:** all-wheel steering, autonomous vehicle, guidance control, heading angle, localization, magnetic marker system, positioning

## Abstract

Real-time continuous localization is a key technology in the development of intelligent transportation systems. In these systems, it is very important to have accurate information about the position and heading angle of the vehicle at all times. The most widely implemented methods for positioning are the global positioning system (GPS), vision-based system, and magnetic marker system. Among these methods, the magnetic marker system is less vulnerable to indoor and outdoor environment conditions; moreover, it requires minimal maintenance expenses. In this paper, we present a position estimation scheme based on magnetic markers and odometry sensors for an all-wheel-steering vehicle. The heading angle of the vehicle is determined by using the position coordinates of the last two detected magnetic markers and odometer data. The instant position and heading angle of the vehicle are integrated with an extended Kalman filter to estimate the continuous position. GPS data with the real-time kinematics mode was obtained to evaluate the performance of the proposed position estimation system. The test results show that the performance of the proposed localization algorithm is accurate (mean error: 3 cm; max error: 9 cm) and reliable under unexpected missing markers or incorrect markers.

## 1. Introduction

Many studies on autonomous navigation systems (ANSs) in intelligent transportation systems (ITSs) have been conducted. It has been determined that information on real-time vehicle position and heading is essential for an ANS. To measure position and heading information, various studies have employed GPS, laser scanners, land marker, and vision systems. GPS is the most commonly used technique for identifying the vehicle position in outdoor environments. Numerous studies have therefore focused on ANS using GPS [1,2,3,4,5,6]. However, GPS for ANS requires costly equipment for high accuracy; furthermore, its positioning performance can be negatively affected by buildings, tunnels, and woods, while its use can be additionally constrained on account of jamming for military purposes. A vision system is applied to detect lanes on roads or to extract the characteristics of surroundings; this information is used to identify the vehicle position or obstacles [7,8,9]. In such cases, vehicle positioning through the image information process may become unstable because of changes in operating conditions, such as day to night, snow to rain, or indoors to outdoors. Therefore, ANS requires a more stable, accurate, and cost-effective technology for positioning. If the driving path of an automatic driving vehicle is determined like that of a tram or bus, a magnetic marker system could be a more effective, semipermanent, and economical alternative to the DGPS system with regard to construction costs [10].

Many studies on ANS have evaluated the magnetic marker system because it is rarely affected by buildings, tunnels, woods, or climatic conditions [11,12,13,14,15,16]. A study combining GPS with the magnetic marker system was proposed to leverage the advantages of each system [17]. The magnetic guidance system applies magnetic materials in the form of magnetic tape or a magnetic marker to the road. It then analyzes the magnetic signal detected when a vehicle equipped with a magnetic detection sensor passes; the system measures the vehicle’s relative position on the road center or its absolute position [18]. This system is resistant to environment conditions, such as light, weather, and temperature, while providing centimeter-level-position accuracy. Binary code information using polarity is available as well. The California Partners for Advanced Transit and Highways (PATH) research and development center developed a guidance control system for a snow plow using the magnetic guidance system [19]. In addition, the Msdar city personal rapid transit system (PRT) [20] adopted automatic guidance control technology using the magnetic marker of the Free Range on Grid (FROG) navigation system [21].

In automated guided vehicles, magnetic-marker-based automatic transportation technology has long been utilized, but these systems have been used at low speeds below 15 km/h and low-floor transportation systems for magnetic signal processing, which is mainly applied under strict magnetic-marker-installation regulations in factories. In order to utilize the magnetic marker system for a public transportation system operating at more than 30 km/h on public roads, the magnetic marker system requires a more precise magnetic signal processing and position estimation technology that can distinguish the magnetic markers from the various magnetic characteristic signals measured from the road surface. In this paper, we propose a position estimation technology that, using magnetic markers, can distinguish between magnetic signals observed while driving on a road, and can safely operate without being affected by driving conditions even when some markers are missing.

Use of the magnetic marker system for ANS requires specific considerations. GPS can provide the absolute position of an antenna on a real-time basis, whereas the magnetic detection sensor provides the detected relative distance between the magnetic marker and magnetic sensing ruler. Thus, a number of technologies should be combined to calculate the absolute position of the vehicle based on the magnetic marker. It is first necessary to identify which of the magnetic markers is the detected one. Second, accurate heading information is required to calculate the absolute position of the vehicle. Third, although the magnetic marker is detected at irregular time intervals depending on the travel speed and magnetic marker installation interval, the position estimation system should be able to continuously calculate the position even in a section or during intervals without magnetic detection. To that end, dead reckoning (DR) [22,23,24], which is based on the vehicle kinematic model, is applied. In conventional studies on position estimation, the position and heading of a vehicle are concurrently estimated by combining the magnetic marker and relative positional information through an extended Kalman filter (EKF). However, because the values are calculated by combining the position and heading, an error can occur if either of them is incorrect [12,13,14,15]. In this study, the absolute position of the all-wheel steering vehicle is estimated based on the magnetic marker.

We therefore present a vehicle position initialization method by searching the polarity arrangement of a magnetic marker for a vehicle whose initial position is unknown. The heading angle is determined by using absolute positional information on the last two magnetic markers detected in sequence during the operation; that is, the magnetic sensor information and odometry information. Once the heading angle is determined, the absolute position of the vehicle is determined using that information and the magnetic sensor detection distance. The heading and absolute position of the vehicle are determined only at the time of the magnetic detection; therefore, this information should be combined with the EKF to calculate the continuous position on a real-time basis.

To validate the proposed method, we verify its performance in a comparison with the position measured with the GPS real-time kinematics (RTK) mode. We present the errors of the position estimation whenever each magnetic marker is detected. The results show that the proposed method can distinguish an invalid marker that may be accidently detected on the road.

The remainder of this paper is arranged as follows: in Section 2, we describe the structure and configuration of a test vehicle and track. In Section 3, we present the proposed position/heading estimation method. In Section 4, we describe the test results with regard to the feasibility of the proposed position/heading estimation method. The study results and our conclusions are provided in Section 5.

## 2. Magnetic Markers-Based Positioning System

**Figure 1:**
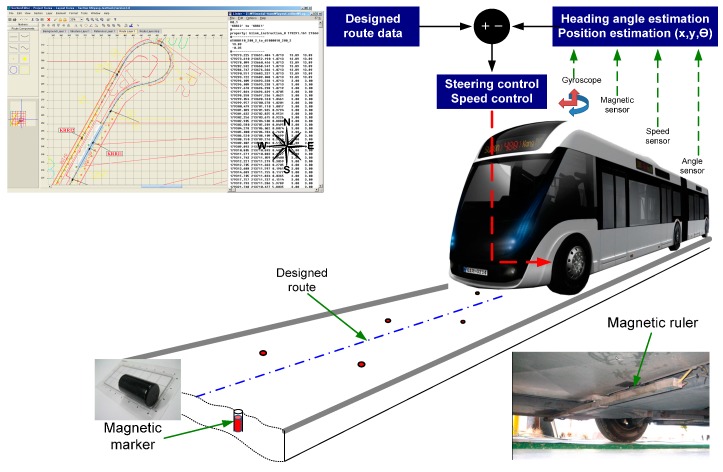
Schematic of automatic guidance system.

The objective of the magnetic marker-based positioning system is to recognize a land marker and accurately measure the vehicle position using that information. When applying the land marker to a positioning system, the features of the land marker and positional information are stored in computer memory. In terms of the magnetic marker, the coordinates and polarities of magnetic markers buried under the road are stored in computer memory. The whole configuration of the magnetic marker-based automatic guidance control system is illustrated in Figure 1. The coordinates of the reference route are stored in an automatic control computer for ANS. This reference route information includes the coordinates of the reference route and the speed limit, heading, and stop position. The magnetic markers are buried under the road; the locations are measured and stored in the control computer.

The on-board positioning system calculates the absolute position and heading of the vehicle using stored information and information measured by sensors installed in the vehicle, including the magnetic detection sensors. The control system manages the speed and position of the vehicle using the discrepancy between the calculated vehicle position and the reference route. The present study is limited to the positioning system for ANS; this section presents the test track configuration and the structure of the on-board magnetic detection system and measurement sensor.

### 2.1. Test Route

**Figure 2:**
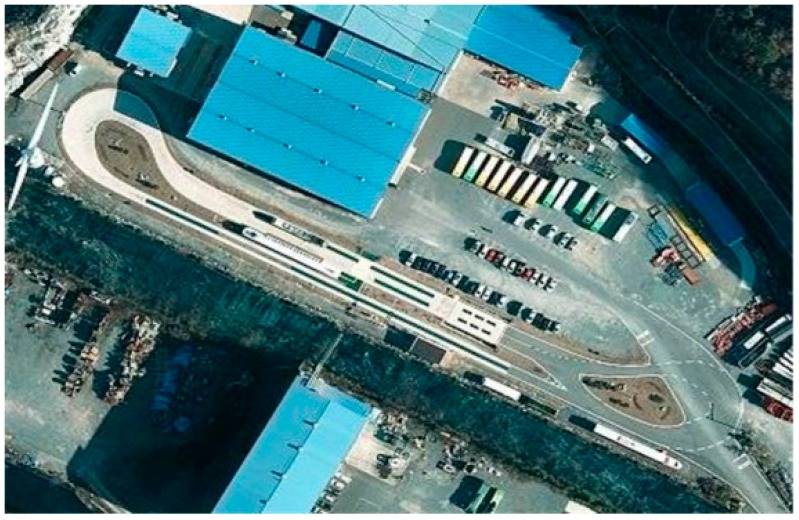
Test track.

The test track is in a loop shape, as shown in Figure 2. The total length is 476 m; magnetic markers are buried along the route. Eleven magnetic markers are buried at intervals of 1 m at two sections for the initialization; other routes are set at intervals of 2 to 5 m. One station and two 12-m-radius turn-arounds exist. A bridge is situated at the center of the test track.

**Figure 3:**
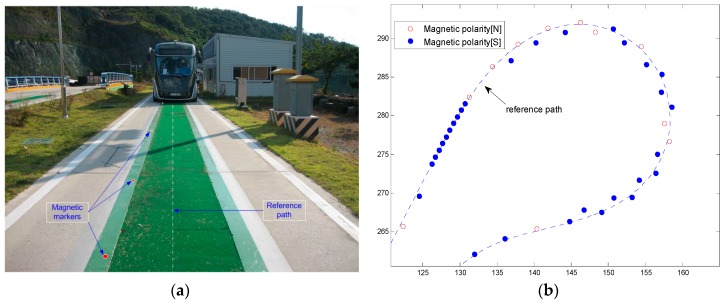
Marker installation on the path. (**a**) Artificial lawn on the path; (**b**) Magnetic markers in the curved section.

A 15-mm-diameter and 30-mm-long cylindrical magnetic marker is installed along the test track. The absolute coordinate of the magnetic marker buried under the road is measured using a precision instrument (±5 mm); it is then recorded. Figure 3a shows the straight section of the test track, where the magnetic markers are concentrated on the left on account of the center artificial turf. Figure 3b shows the curved section of the test track where the magnetic markers are buried in a zigzag manner to enhance the detection rate. Table 1 shows the coordinate data of the magnetic markers used for the position detection.

### 2.2. Magnet Measurement System

**Figure 4:**
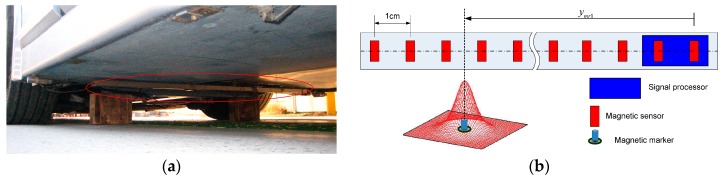
Magnetic ruler. (**a**) Installation; (**b**) Configuration.

The magnetic ruler of the FROG system is used as a major sensor of the position detection system [21]. The magnetic sensor is installed on the vehicle sub-frame, as shown in Figure 4a. When passing the magnetic marker buried under the road, it analyzes the detected magnetic signal. In addition, it measures the detection position of the magnetic marker and the polarity information, which are then transmitted to the control computer.

The magnetic ruler is structured as depicted in Figure 4b; its specifications are outlined in Table 2. To analyze the detected magnetic signals, the magnetic ruler includes magnetic sensors installed at 1-cm intervals and the processor. The processor calculates the center position of the detected magnetic markers using the signal values of the sensors.

### 2.3. Vehicle Measurement System

**Figure 5:**
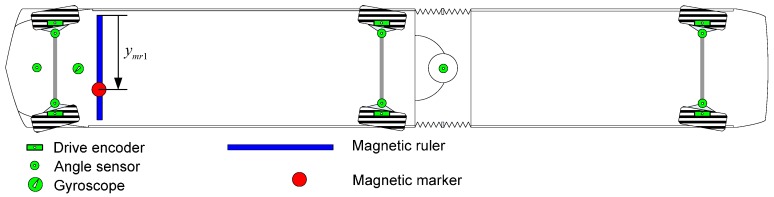
Sensors installed in the test vehicle.

The test vehicle is a triaxial, six-wheel, articulated vehicle with a low floor and rubber tires with a length of 18 m, as shown in Figure 5. This vehicle is designed to have three independent steering axles and a 12-m minimum turning radius. The vehicle is equipped with various sensors, such as a wheel encoder, steering angle sensor, magnetic ruler, and gyroscope.

## 3. Magnetic Marker-Based Position and Heading Estimation

To accomplish effective guidance control, the lateral tracking error of each carriage must be accurately measured. Therefore, the position and heading angle of the vehicle must be obtained in real time. This section presents an algorithm for estimating the position and heading angle. The heading of the vehicle is calculated in the moment of detection of the magnetic marker by the magnetic ruler. This value is combined with the gyroscope data; the continuous heading of the vehicle is then calculated. The position of the vehicle at the moment of magnetic detection is determined based on this value. The continuous absolute position of the vehicle is estimated by combining the measured position with EKF.

### 3.1. Heading Estimation

**Figure 6:**
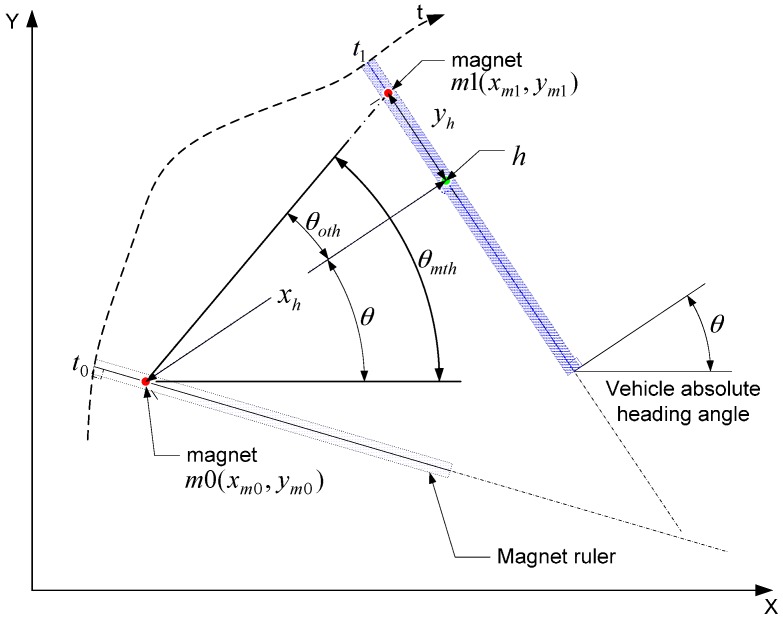
Concept of heading angle estimation.

The heading of the vehicle in the magnetic marker detection system can be calculated only when the magnetic ruler is detecting the magnetic marker. Assume that the perpendicular point to the magnetic ruler detecting *m*1 at *t*_1_, from the position of *m*0 detected at *t*_0_, is *h*. When the magnetic ruler of the vehicle detects magnetic markers (*m*0,*m*1) in sequence, as shown in Figure 6, and coordinates of the magnetic markers are known, the absolute heading of the vehicle at the time of detecting the second magnetic marker (*t*_1_) can be determined when the distances (*x_h_*,*y_h_*) from the each magnet (*m*0,*m*1) to *h* are known. That is, *θ_mth_* is the absolute heading angle of the straight line linking the two magnets (*m*0,*m*1), which can be calculated as:

(1) $$\theta_{mth}=\mathrm{atan\sim}2\left(y_{m1}-y_{m0},\;x_{m1}-x_{m0}\right)$$

where (*x_m_*_0_,*y_m_*_0_) and (*x_m_*_1_,*y_m_*_1_) are the absolute positions of each magnet.

*θ_oth_* is the changed angle of the vehicle until the moment of detecting the second magnet from the first detection by a turn motion. If *x_h_*_1_ and *y_h_*_1_ are known, this can be calculated as:

(2) $$\theta_{oth}=\mathrm{atan}\left(\frac{y_{h}}{x_{h}}\right)$$

Thus, the instant absolute heading angle (*θ_i_*) of the vehicle at time *t*_1_ detecting the magnet (*m*1) can be obtained from the difference between the magnetic heading (*θ_mth_*) and turn angle of the vehicle (*θ_oth_*), as shown in [10].

(3) $$\theta_{i}=\theta_{mth}-\theta_{oth}$$

### 3.2. Position Estimation

The magnetic marker buried under the road is not identified when detected; therefore, a method to identify the detected magnet is necessary. To address this need, magnetic markers are buried at specific intervals and in a unique polarity array at a designated section. The magnet is identified in such a way that the polarity array of the magnetic marker collected in sequence while the vehicle is traveling on the road is searched in a magnet database to obtain the coincident section and identify the last detected magnet. When the detected magnet is identified, the initial heading and position are determined using the identified magnet information; then, the continuous position of the vehicle is determined by EKF combined with the vehicle kinematic model.

#### 3.2.1. Magnetic Marker Identification and Position Initialization

The vehicle’s position is unknown until it passes the initialization section; thus, the detected magnetic marker cannot be identified. Consequently, an initialization process to identify the detected magnet is required. As a means of identifying the detected magnets, the positioning system collects 11 magnets detected at 1-m intervals at the initialization section while the vehicle is traveling. The magnetic marker database in the positioning system contains the magnet positional information from along the road and the magnetic polarity pattern information in the initialization section. It thus seeks a polarity array pattern of 11 magnets detected by the magnetic ruler in the magnetic marker database. When the interval is 1 m and 11 polarity patterns are uniquely coincident, the last-detected magnetic marker becomes identifiable. Once the magnetic marker is identified, the coordinates of the magnetic marker ($x_{m},y_{m}$) can be obtained from the magnet database.

**Figure 7:**
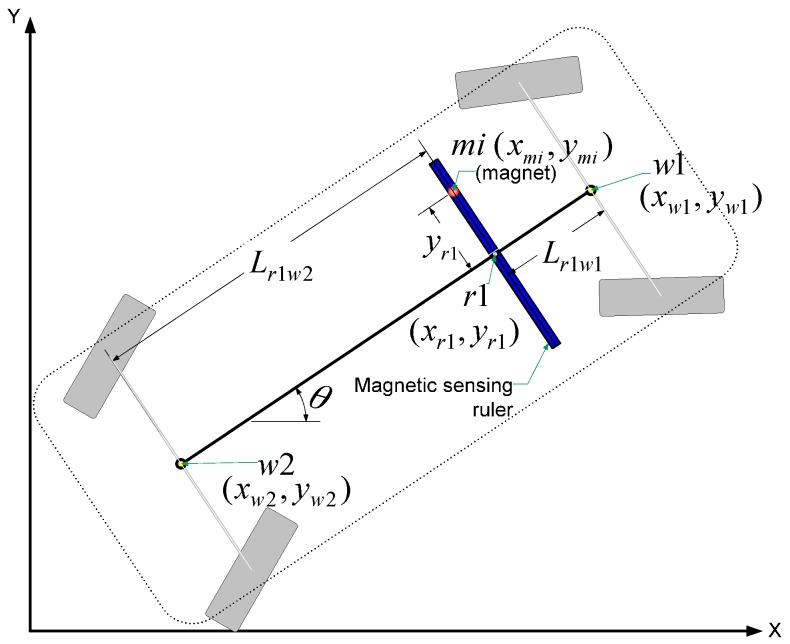
Vehicle configuration and measurement model.

Once the coordinates of the detected magnetic marker are known, the vehicle heading can be determined according to Equations (1)–(3). As shown in Figure 7, when the position (*x_mi_*,*y_mi_*) of the detected magnet, the distance (*y_r_*_1_) measured by the magnetic ruler, and the vehicle heading angle (*θ*) are known, the position of the rear wheel center (*w*2) can be determined according to:

(4) [xw2yw2]=[xmiymi]+yr1[sin θ−cos θ]−Lr1w2[cos θsin θ]

where (*x_mi_*,*y_mi_*) and (*x_m_*_2_,*y_m_*_2_) are the respective absolute positions of the detected magnet and rear wheel center. In addition, *y_r_*_1_ is the distance from the magnetic ruler center to the detection point, *L_r_*_1*w*2_ is the distance between the rear wheel center (*w*2) and magnetic ruler center (*r*1), and *θ* is the vehicle heading.

#### 3.2.2. Integration of the Magnetic Marker and Sensor Using EKF

Position correction is possible only when detecting the magnetic marker. Magnetic marker detection is possible only when the vehicle passes the magnetic marker. Accordingly, in terms of time, position correction is discontinuously and irregularly performed. Therefore, the magnetic detection information and EKF are combined to obtain regular and continuous positional information from such irregular information.

**Figure 8:**
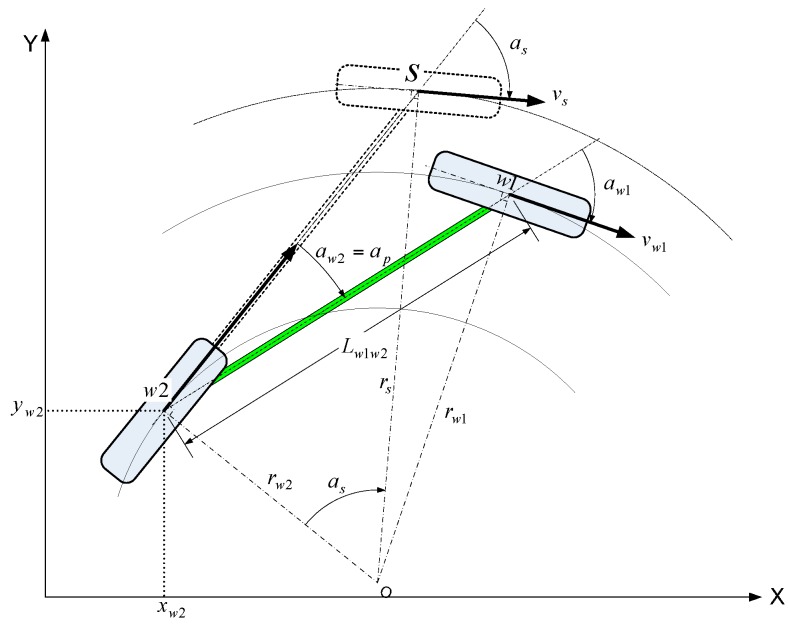
All-wheel steering bicycle model.

Vehicle model: A propulsion motor is mounted on axles 2 and 3, whereas axle 1 has non-powered wheels. Accordingly, compared to a model using the wheel speed of axle 2, the vehicle kinematic model using the wheel speed of axle 1 can minimize vehicle modeling errors, such as a tire slip generated by the power wheel. Thus, the all-wheel steering model is converted to a front-wheel steering model for the vehicle model used in EKF. This relationship is expressed in Figure 8. A bicycle model is applied to simplify the model. The distance between the rear wheel center (*w*2) and front wheel center (*w*1) is represented by *L_w_*_1*w*2_, and the steering angles of the front and rear wheel are described by *a_w_*_1_ and *a_w_*_2_, respectively. Additionally assume that the vehicle moves around a virtual central axle (*O*). Therefore, a new virtual front-wheel axle center (*s*) is established to convert the all-wheel steering to a front-wheel steering system. *s* is the virtual point at distance *L_w_*_1*w*2_ in a straight line to the rear-wheel steering angle (*a_w_*_2_) from the rear-wheel central axle (*w*2). This is intended to obtain the variables (*v_s_*,*a_s_*,*a_p_*) of motion Equations (11)–(13) to produce the same momentum as thing of *w*2 generated by the all-wheel and front-wheel steering models, respectively.

In this case, *v_s_* is the speed of the front wheel on virtual axle *s*, *a_s_* is the front-wheel steering angle on the virtual axle, and *a_p_* is the rear-wheel steering angle of the all-wheel steering vehicle. Then, variables that lead to the same motional relations at common point *w*2 of the virtual and actual axles are obtained as described below.

First, the angular speed (*ω_w_*_1_) at *w*1 in Figure 8 shall be the same as angular speed (*ω_s_*) at *s*. Accordingly, if angular speed (*ω_w_*_1_) and (*ω_s_*) are equal, the virtual variables (*v_s_*,*a_s_*,*a_p_*) at the virtual front-wheel axle center (*s*) are obtained according to:

(5) $$v_{s}=v_{w1}\frac{r_{s}}{r_{w1}}$$

(6) $$a_{s}=\mathrm{atan}\left(\frac{L_{w1w2}}{r_{w2}}\right)$$

(7) $$a_{p}=a_{w2}$$

The radius from the rotation axis center (*O*) to surrounding points is obtained using trigonometry according to:

(8) $$r_{w1}=L_{w1w2}\frac{\cos\left(a_{w2}\right)}{\sin\left(a_{w1}-a_{w2}\right)}$$

(9) $$r_{w2}=L_{w1w2}\frac{\cos\left(a_{w1}\right)}{\sin\left(a_{w1}-a_{w2}\right)}$$

(10) $$r_{s}=sign\left(a_{s}\right)\sqrt{r_{w2}^{2}+L_{w1w2}^{2}}$$

where if x < 0,sign(x) is −1; otherwise, *sign*(*x*) is +1.

Thus, the motion equation of the all-wheel steering vehicle is obtained according to:

(11) $${\dot{x}}_{w2}=v_{s}\cos(a_{s})\cos\left(a_{p}\right)$$

(12) $${\dot{y}}_{w2}=v_{s}\cos(a_{s})\sin\left(a_{p}\right)$$

(13) $$\dot{\theta }=\frac{v_{s}}{L_{w1w2}}\sin(a_{s})$$

(14) $$\Delta x\left(k\right)={\dot{x}}_{w2}\;\Delta t$$

(15) $$\Delta y\left(k\right)={\dot{y}}_{w2}\;\Delta t$$

(16) $$\Delta \theta \left(k\right)=\dot{\theta }\;\Delta t$$

(17) $$\left[\begin{array}{c} x_{w2}\left(k\right)\\ y_{w2}\left(k\right) \end{array}\right]=\left[\begin{array}{c} x_{w2}\left(k-1\right)\\ y_{w2}\left(k-1\right) \end{array}\right]+Rot(\theta (k-1))\left[\begin{array}{c} \Delta x\left(k\right)\\ \Delta y\left(k\right) \end{array}\right]$$

(18) θ(k)=θ(k−1)+Δθ(k)

(19) $$Rot(\theta (k-1))=\left[\begin{array}{cc} \cos\left(\theta \left(k-1\right)\right) & -\sin\left(\theta \left(k-1\right)\right)\\ \sin\left(\theta \left(k-1\right)\right) & \cos\left(\theta \left(k-1\right)\right) \end{array}\right]$$

where (*x_w_*_2_,*y_w_*_2_) is the position of rear-wheel center *w*2 of the all-wheel steering vehicle, *θ* is the vehicle heading, and Δ*t* is the sampling time.

Based on the above equation, the position of the vehicle at the sampling time can be estimated using the given initial position of the vehicle and the heading information, front-wheel axis speed (*v_w_*_1_), front-wheel steering angle (*a_w_*_1_), and rear-wheel steering angle (*a_w_*_2_).

**Figure 9:**
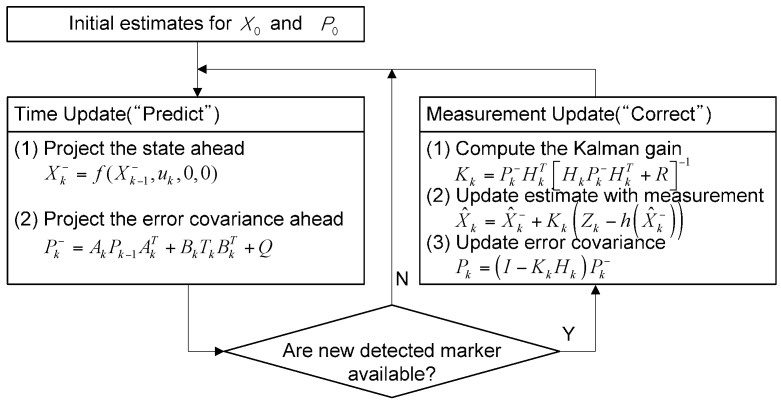
EKF flow chart.

EKF comprises the time update and measurement update for the vehicle positioning. The time update is regarded as the position prediction stage, while the measurement update is considered the correction stage. The position correction is implemented only when measuring the magnetic marker. The work flow is depicted in Figure 9.

System Model: Equations (17)–(19) are used for the system model, and state vector *X_k_* = [*x_w_*_2_(*k*)*y_w_*_2_(*k*)*θ*(*k*)]*^T^* and input *u_k_* = [Δ*x*(*k*)Δ*y*(*k*)Δ*θ*(*k*)]*^T^* are applied. This can be simplified according to Equation (20):

(20) $$X_{k}=f(X_{k-1},u_{k},\gamma_{k},\sigma_{k})$$

where *γ_k_* and *σ_k_* respectively denote the system and input noise with covariance matrices *Q* and *T_k_*.

Measurement Model: The central position of axle 2 and the heading are defined by the measurement when detecting the magnetic marker according to Equation (21):

(21) $$Z_{k}=h\left(X_{k},k\right)+v_{k}={\left[x_{mw2}(k)\;y_{mw2}(k)\;\theta (k)\right]}^{T}$$

where $v_{k}$ is considered the zero-mean white noise with covariance matrix R, which is known as the measurement noise.

Prediction Stage [25,26]: The system future state and the future state of the state error covariance matrix are predicted using the time update equation:

(22) $$X_{k}^{-}=f(X_{k-1}^{-},u_{k},0,0)$$

(23) $$P_{k}^{-}=A_{k}P_{k-1}A_{k}^{T}+B_{k}T_{k}B_{k}^{T}+Q$$

where *A_k_* and *B_k_* are Jacobian matrices to the partial differential of *f*(·) to *X_k_* and *u_k_*:

(24) $$A_{k}=\left[\begin{array}{ccc} \frac{\partial f_{x}}{\partial x_{w2}} & \frac{\partial f_{x}}{\partial y_{w2}} & \frac{\partial f_{x}}{\partial \theta }\\ \frac{\partial f_{y}}{\partial x_{w2}} & \frac{\partial f_{y}}{\partial y_{w2}} & \frac{\partial f_{y}}{\partial \theta }\\ \frac{\partial f_{\theta }}{\partial x_{w2}} & \frac{\partial f_{\theta }}{\partial y_{w2}} & \frac{\partial f_{\theta }}{\partial \theta } \end{array}\right]=\left[\begin{array}{ccc} 1 & 0 & -\Delta x(k)\sin(\theta (k))-\Delta y(k)\cos(\theta (k))\\ 0 & 1 & \Delta x(k)\cos(\theta (k))-\Delta y(k)\cos(\theta (k))\\ 0 & 0 & 1 \end{array}\right]$$

(25) $$B_{k}=\left[\begin{array}{ccc} \frac{\partial f_{x}}{\partial \Delta x} & \frac{\partial f_{x}}{\partial \Delta y} & \frac{\partial f_{x}}{\partial \Delta \theta }\\ \frac{\partial f_{y}}{\partial \Delta x} & \frac{\partial f_{y}}{\partial \Delta y} & \frac{\partial f_{y}}{\partial \Delta \theta }\\ \frac{\partial f_{\theta }}{\partial \Delta x} & \frac{\partial f_{\theta }}{\partial \Delta y} & \frac{\partial f_{\theta }}{\partial \Delta \theta } \end{array}\right]=\left[\begin{array}{ccc} \cos(\theta (k)) & -\sin(\theta (k)) & 0\\ \sin(\theta (k)) & \cos(\theta (k)) & 0\\ 0 & 0 & 1 \end{array}\right]$$

The system position noise and heading noise standard deviations were taken to be 0.03 m and 0.3°, respectively. Therefore, the system covariance matrix:

(26) $$Q=\left[\begin{array}{ccc} 0.03^{2} & 0 & 0\\ 0 & 0.03^{2} & 0\\ 0 & 0 & 0.00523^{2} \end{array}\right]$$

The input noise standard deviation was taken to be 0.052 m and 0.0025° considering the sensor specifications and uncertain wheel radius, respectively. Thus, the input noise covariance matrix:

(27) $$T=\left[\begin{array}{ccc} 0.052^{2} & 0 & 0\\ 0 & 0.052^{2} & 0\\ 0 & 0 & 0.000043^{2} \end{array}\right]$$

Correction Stage: The correction stage is implemented only when the effective magnetic marker is detected. The Kalman gain matrix, state estimate, and state error covariance matrix for the updated state estimate are calculated as follows:

(28) $$K_{k}=P_{k}^{-}H_{k}^{T}{\left[H_{k}P_{k}^{-}H_{k}^{T}+R\right]}^{-1}$$

(29) $${\hat{X}}_{k}={\hat{X}}_{k}^{-}+K_{k}\left(Z_{k}-h\left({\hat{X}}_{k}^{-}\right)\right)$$

(30) $$P_{k}=\left(I-K_{k}H_{k}\right)P_{k}^{-}$$

where *I* is the identity matrix and *H_k_* denotes the Jacobian matrices of the partial differential of *h*(·)to *X_k_*:

(31) $$H_{k}=\left[\begin{array}{ccc} \frac{\partial h_{x}}{\partial \Delta x} & \frac{\partial h_{x}}{\partial \Delta y} & \frac{\partial h_{x}}{\partial \Delta \theta }\\ \frac{\partial h_{y}}{\partial \Delta x} & \frac{\partial h_{y}}{\partial \Delta y} & \frac{\partial h_{y}}{\partial \Delta \theta }\\ \frac{\partial h_{\theta }}{\partial \Delta x} & \frac{\partial h_{\theta }}{\partial \Delta y} & \frac{\partial h_{\theta }}{\partial \Delta \theta } \end{array}\right]\;=\left[\begin{array}{ccc} 1 & 0 & 0\\ 0 & 1 & 0\\ 0 & 0 & 1 \end{array}\right]$$

The state error noise covariance was taken to be 1 as uncertain start position error as follows:

(32) $$P_{0}=\left[\begin{array}{ccc} 1 & 0 & 0\\ 0 & 1 & 0\\ 0 & 0 & 1 \end{array}\right]$$

The measurement noise standard deviation was taken to be 0.01 m and 0.5° as specifications of the measured data quality for a marker position. Thus, the measurement noise covariance matrix:

(33) $$R=\left[\begin{array}{ccc} 0.01^{2} & 0 & 0\\ 0 & 0.01^{2} & 0\\ 0 & 0 & 0.00872^{2} \end{array}\right]$$

#### 3.2.3. Identification of the Valid Magnetic Marker and Positioning Error

When detecting the magnetic marker while the vehicle is moving, the coordinate of the magnetic marker is estimated according to:

(34) [x^miy^mi]=[x^w2y^w2]−yr1[sin θ−cos θ]−Lr1w2[cos θsin θ]

The coordinate of the nearest magnet marker with the estimated marker can be obtained by calculating the Euclidean distance between the estimated marker position and the marker positions in the database according to:

(35) $$(i_{\min},d_{\min})=\min\left(\sqrt{{\left(X_{DB}-{\hat{x}}_{mi}\right)}^{2}+{\left(Y_{DB}-{\hat{y}}_{mi}\right)}^{2}}\right)$$

where *i*_min_ is the index number of the nearest magnet marker in the database, *d*_min_ is the Euclidean distance to the nearest magnet marker, and (*X_DB_*,*Y_DB_*) denotes the X and Y coordinates of the magnet marker in the database, respectively. Moreover, the distance to the nearest magnetic marker, *d*_min_, is used as the positioning error indicator.

In this study, the distance within 20 cm is considered to detect the valid magnetic marker, and the position of the magnetic marker ($x_{i_{\min}},y_{i_{\min}})$ is used. When minimum distance *d_min_* is more than 20 cm, the detected magnetic signal is neglected. The adoption of such an approach distinguishes the valid marker from the detected marker. The 20-cm guideline is applied in this study because it was determined that the estimation error from the marker positioning while traveling is less than 10 cm.

## 4. Experimental Results: Position Estimation Performance

### 4.1. Positioning System for Performance Evaluation

**Figure 10:**
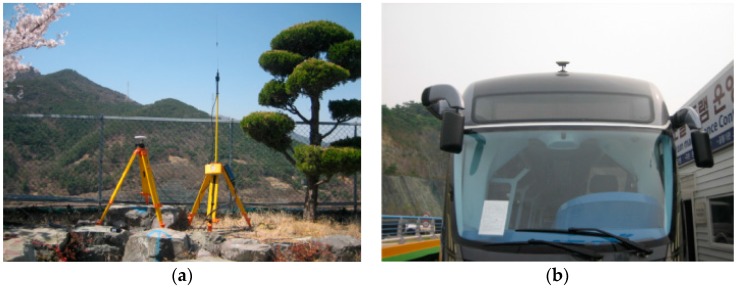
GPS measurement. (**a**) GPS base station; (**b**) Rover GPS on the vehicle.

To measure the absolute position of the traveling vehicle, two GPS receivers (X60 GPS, RTK: accuracy ±20 mm; 5-Hz sampling rate) were used. One was installed at a known position on the ground, as shown in Figure 10a, and it was used as a base station; the other was mounted on the top of the center of axle 1 of the vehicle, as depicted in Figure 10b.

### 4.2. Vehicle Positioning Test

#### 4.2.1. Positioning Test Result

**Figure 11:**
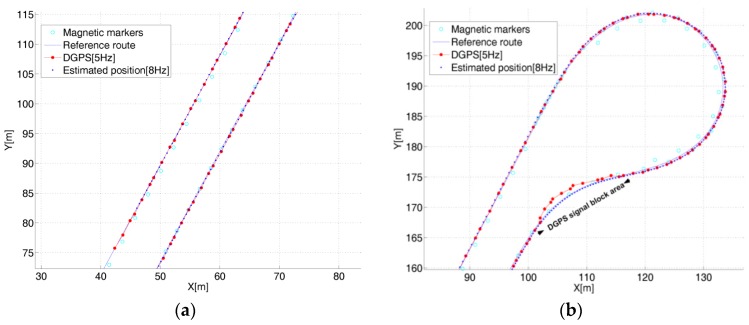
GPS position and estimated position. (**a**) Position estimation on the straight route; (**b**) Position estimation on the upside curve.

The positioning performance of the proposed algorithm was compared to the GPS-measured values. The error could not be directly calculated by point-to-point matching because the frequency of the estimation system (8 Hz) and GPS frequency (5 Hz) were not coincident. Thus, the performance evaluation was replaced with the verification of the coincident trajectory by the GPS position and estimated position, as shown in Figure 11a,b. The position of the magnetic marker and the reference travel route, GPS position, and estimated position were presented together as a result of the test. The positioning trajectory measured by GPS and the estimated position trajectory were coincident on the straight and curved routes. As shown in Figure 11b, a significant error between the GPS position and estimated position occurred for a portion of the route. This error was attributed to the poor performance of GPS due to the station or surrounding buildings on some curved sections of the test track.

#### 4.2.2. Magnetic Marker Detection and Validity Judgment

**Figure 12:**
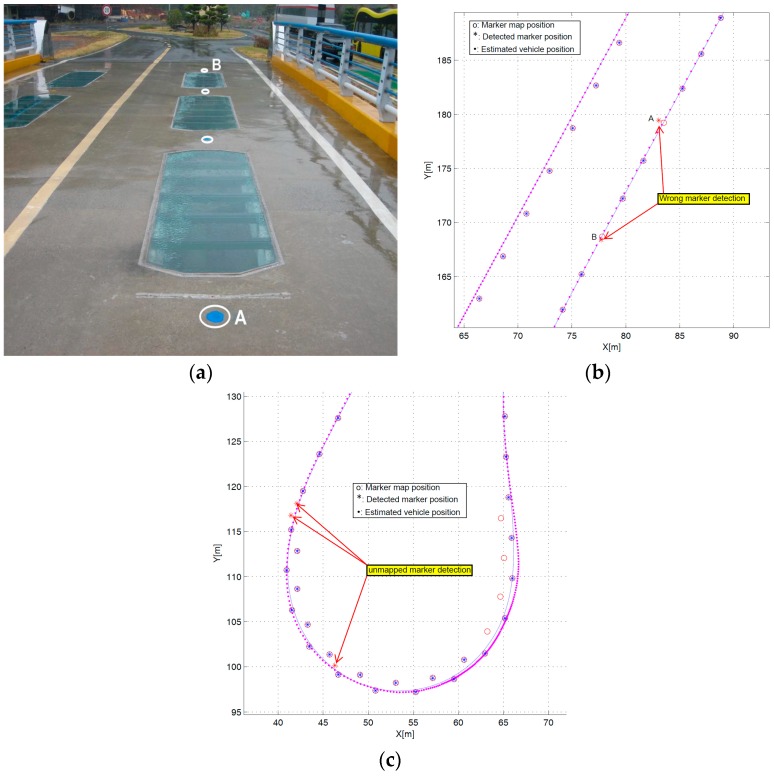
Marker detection and position estimation. (**a**) Magnetic markers on the bridge; (**b**) Marker detection fails on the bridge; (**c**) Unmapped marker detection in the curved section.

The positioning system calculated the error between the actual and estimated position of magnetic markers when detecting the magnetic marker. The algorithm only allowed magnetic markers with an estimated error of less than 20 cm. The positioning system determined the validity of the detected magnetic marker using this information. While moving, the vehicle may detect magnetic signals other than the magnetic marker, which means there could be detection of a signal interference environment, such as a closed circuit installed along the road for traffic detection, the steel structure of a bridge, or a manhole cover. The steel structure of a bridge was situated on the test track. Figure 12a shows the bridge and magnetic marker position on the test track.

Figure 12b illustrates the magnetic detection error result at two positions on the bridge (A, B). At these two locations, the detected markers were discarded to exclude inappropriate magnetic detection information because the difference between the actual magnetic marker position (o) and the estimated position (*) exceeded the assigned limit by 20 cm. Such an error was attributable to magnetic interference by the bridge’s steel structure.

**Figure 13:**
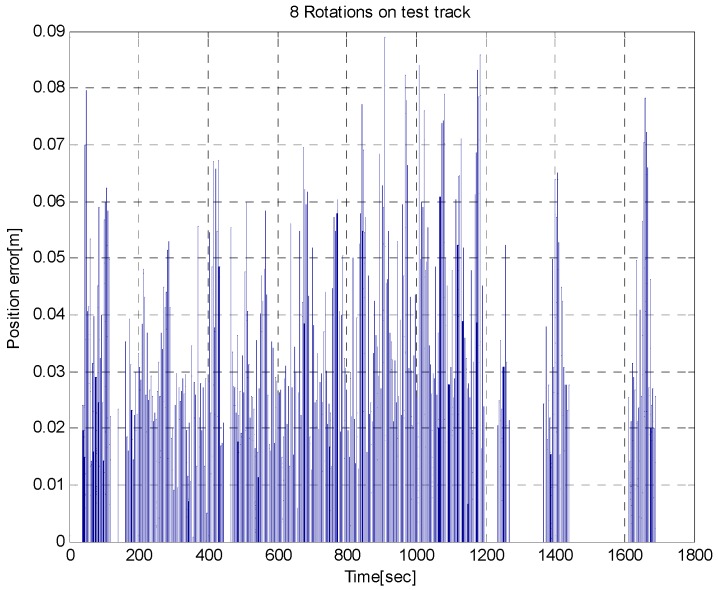
Position error based on the marker during eight test laps.

As shown in Figure 12c, three neglected magnetic markers were detected in the curved section. These were the magnetic markers installed for another path; however, they were not included in the magnetic marker database and were thus neglected. Based on the above test result, the validity of the magnetic signals detected during traveling could be determined by the proposed positioning system. This result demonstrates the advantage of the proposed positioning algorithm against external interference. In addition, Figure 13 shows that the position errors were found to be approximately 0.03 m, on average, with a maximum error of 0.089 m during eight test laps in the test track.

## 5. Conclusions

Localization in ITS is a very critical function. In this paper, we have presented a position estimation scheme based on magnetic markers and odometry sensors for a vehicle with 18-m-long articulation and three-wheel steering. The instant heading angle of the vehicle is determined by using the position coordinates of the last two detected magnetic markers and odometer data. The instant position and heading angle of the vehicle are integrated with an extended Kalman filter to estimate the continuous position. GPS data in the real-time kinematics mode was obtained to evaluate the performance of the proposed position estimation system.

An important function of the magnetic marker-based positioning system is to calculate the vehicle’s absolute position (latitude, longitude) based on relative positional information, polarity information measured by a magnetic ruler, and an odometry sensor during traveling. To that end, we proposed a method to determine the:

- Initial position and heading of the vehicle using the polarity array of the magnetic marker when the vehicle position is unknown.
- Continuous positioning combined with EKF using the instant position and heading at the moment of magnetic marker detection.
- To verify the validity of the proposed method, we compared its estimated position results with those using on-board GPS data. To verify the positioning performance under an actual environment without GPS, an evaluation of the positioning performance was conducted based on estimated magnetic marker positional information. The proposed algorithm showed superior performance in a magnetic interference environment on the road.

## Figures and Tables

**Table 1:** Magnetic marker database [10].

Index	Magnetic Marker Position		Polarity (N = 2, S = 1)
	$x_{m}$ [m] (E)	$y_{m}$ [m] (N)	
1	179,296.216	213,693.823	1
2	179,296.684	213,694.693	2
3	179,297.179	213,695.559	2
4	179,297.658	213,696.437	1
5	179,298.147	213,697.315	2

**Table 2:** Specifications of the magnetic ruler [21].

Magnetic Sensor Array Gap	1 cm
Horizontal measurement range	32 to 256 cm
Measurement height allowable	5 to 40 cm (from ground)
Degree of precision	±2 cm (H 20 cm, speed 10 m/s)
Operation temperature range	−20 to 70 °C
Maximum speed range	25 m/s (90 km/h)
